# Intimate Partner Violence and HIV Outcomes Among Women Living with HIV in Durban, South Africa

**DOI:** 10.1007/s10461-024-04318-x

**Published:** 2024-06-13

**Authors:** Sheila O. Ojeaburu, Jienchi Dorward, Lauren R. Violette, Andrew Gibbs, Hlengiwe Shozi, Yukteshwar Sookrajh, Thobile Mhlongo, Hope Ngobese, Nigel Garrett, Paul K. Drain

**Affiliations:** 1 Centre for the AIDS Programme of Research in South Africa (CAPRISA), University of KwaZulu–Natal, Durban, South Africa; 2 Department of Medicine, University of California at San Francisco, San Francisco, CA USA; 3 Nuffield Department of Primary Care Health Sciences, University of Oxford, Oxford, UK; 4 Department of Allergy & Infectious Diseases, School of Medicine, University of Washington, Seattle, USA; 5 Department of Epidemiology, School of Public Health, University of Washington, Seattle, USA; 6 South African Medical Research Council, Gender and Health Research Unit, Durban, South Africa; 7 Centre for Rural Health School of Nursing and Public Health, University of KwaZulu-Natal, Durban, South Africa; 8 Institute for Global Health, University College, London, UK; 9 Prince Cyril Zulu Communicable Disease Centre, eThekwini Municipality, Durban, South Africa; 10 School of Nursing and Public Health, Discipline of Public Health Medicine, University of KwaZulu-Natal, Durban, South Africa; 11 Department of Global Health, School of Public Health, University of Washington, Seattle, USA; 12 Department of Global Health, Schools of Medicine and Public Health, University of Washington, Seattle, USA

**Keywords:** Intimate partner violence, Women living with HIV engaged in care, HIV disclosure to partner

## Abstract

We examined the impact of past-year intimate partner violence (IPV) on HIV outcomes among women living with HIV (WLHIV) in Durban, South Africa. We assessed past-year IPV using the WHO Violence Against Women Questionnaire. We conducted logistic regression to assess associations between demographic variables and IPV at baseline, and between IPV at baseline and longitudinal HIV outcomes. Among 235 WLHIV, 17% reported past-year emotional, physical, or sexual IPV. At baseline, HIV-disclosure to partner was associated with 4.35-fold odds of past-year IPV (95% CI 1.17–16.10) after controlling for children, education, and harmful alcohol use. In the prospective analysis, IPV was associated with not achieving the co-primary outcome of retention in care and viral suppression in univariate (OR = 2.32, 95% CI 1.04–5.18), but not in the multivariate model. In the context of rapid treatment scale-up, the high burden of IPV among WLHIV needs to be prioritized, with an emphasis on disclosure support.

## Introduction

While the burden of HIV/AIDS remains high, [1–5] the United Nations’ 95–95–95 campaign articulates a set of global benchmarks to curb the epidemic and strengthen linkage to HIV care [6]. Intimate partner violence (IPV) may result in worse HIV-related health outcomes for women living with HIV (WLHIV) and engaged in HIV care, resulting in decreased well-being and undermining efforts to achieve the UN 95–95–95 targets.

IPV refers to physical, emotional, and/or sexual violence, experienced within intimate relationships [7]. WLHIV with a history of IPV have been found to have poorer health outcomes in general, and HIV-related outcomes, compared to women living with HIV without a history of IPV [8]. Specifically, a history of IPV has been associated with lower antiretroviral therapy (ART) use and engagement in HIV care [8, 9], lower ART adherence, lower viral load (VL) suppression, reduced T-cell function [10–12] and faster progression to AIDS [8, 13]. In these studies, IPV may impact engagement in HIV care, thereby reducing ART adherence, and leading to worse treatment outcomes. These studies are often limited by a small sample size, the use of cross-sectional methodology, and the potential for selection bias; there is also a lack of studies in sub-Saharan Africa.

HIV acquisition, and specifically HIV-disclosure, is also associated with an increased likelihood of experiencing IPV [14, 15], which has wide-ranging physical and psychosocial consequences [12, 16]. Studies on how disclosure of HIV status may impact treatment outcomes for WLHIV and engaged in care are limited.

South Africa bears a significant and disproportionate burden of both HIV and IPV [17]. Population-based studies suggest that past-year IPV prevalence may range from 13 to 20% [18]. However, evidence on the impact of IPV on HIV treatment outcomes is limited [17, 19].

## Objectives

We aimed to estimate the prevalence of past-year IPV in a cohort of WLHIV in Durban, South Africa; to identify sociodemographic factors associated with past-year IPV at baseline; and to examine associations between past-year IPV at baseline and HIV-related health outcomes at 12 months. For the latter analysis, the primary outcome was a composite outcome consisting of viral load suppression (≤ 200 copies/mL) and retention in care at 12 months.

## Methods

### Study Design

This was a secondary, *post-hoc* analysis nested within the STREAM (Simplifying HIV TREATmeant and Monitoring) Study, a single-site, open label randomized control trial of point of care (POC) viral load testing and task shifting based in Durban, South Africa [20, 21]. Male and non-pregnant female adults (aged 18 years or older) living with HIV and on ART for 6 months (N = 390) were enrolled in the study.

Participants were randomized to receive either the standard of care laboratory-based HIV VL monitoring with care provided by professional nurses or doctors, or POC VL monitoring and task-shifting of care to enrolled or professional nurses [22].

We conducted an analysis of all 235 women enrolled in either arm of the STREAM study. Participants provided informed consent and received small reimbursements.

### Study Setting

All study activities occurred at the Centre for the AIDS Programme of Research in South Africa (CAPRISA) eThekwini Clinical Research Site and the adjoining Prince Cyril Zulu Communicable Disease Centre (PCZ CDC), a government funded healthcare clinic in Durban, KwaZulu-Natal, South Africa [22, 23]. Located in Durban’s business district, a major urban and transport hub, the PCZ CDC cares for people living with HIV, as well as individuals with tuberculosis. The clinic has offered ART to all persons living with HIV, regardless of CD4 count since September 2016 [24]. In addition, PCZ CDC offers primary care services in reproductive health, chronic conditions, and other minor illnesses. Female patients presenting with current or past IPV, may be assigned to a social worker with additional counseling training; referrals may be made to a local domestic violence non-profit organization.

### Study Procedures

At enrollment into STREAM, we assessed participants’ experiences of past-year emotional, physical and sexual IPV using the World Health Organization’s Violence Against Women Questionnaire [26]. Participants were asked, “In the past 12 months how many times has…” a particular IPV-related experience occurred. Potential responses included, *never, once, few, many,* or *refused*. Emotional IPV consists of five items; a sample question is, “how many times has a current or previous husband or boyfriend insulted you or made you feel bad about yourself?” Physical IPV consists of five items; a sample question was, “how many times has a current or previous husband or boyfriend ever slapped you or thrown something at you which could hurt you?” Sexual IPV has three items; a sample question was, “how many times has a current or previous husband or boyfriend ever physically forced you to have sex when you did not want to?” Each form of IPV was recoded to either: never (0), or any positive response (1).

Harmful drinking was assessed using the validated Alcohol Use Disorder Identification Test (AUDIT-C) questionnaire (three items; scoring range 0–12; Cronbach alpha 0.84) [24, 25]; scores at least greater than three were considered positive screens [26, 27]. Depression was assessed using the Patient Health Questionnaire-2 (PHQ-2) (two items; scoring range 0–6; Cronbach alpha 0.77); scores at least greater than three were considered positive screens [28, 29].

### Dependent Variables

The primary outcomes were clinically significant markers of HIV-health outcomes. The primary longitudinal analysis examined whether past-year IPV exposure at baseline was associated with the primary outcome for the STREAM study: a composite outcome of viral load (VL) suppression (≤ 200 copies/mL) and retention in care at 12 months from enrollment (i.e., 18 months after ART initiation); these endpoints were also individually analyzed as separate outcome variables. Retention in care was defined as documented pick-up of ART within the 12-month visit window.

### Statistical Analysis

We first described the sample in terms of the frequency of sociodemographic and health-related factors, by IPV-history. For each outcome, we used Chi-squared tests for independence to compare the frequency of relevant outcomes. We then created two logistic regression models. The first model identified baseline sociodemographic factors associated with past-year IPV; here, IPV was evaluated as an outcome. The multivariate model was then adjusted for sociodemographic factors associated with IPV in both the univariate model and in the literature. The second logistic regression model examined associations between past-year IPV at baseline and HIV care outcomes at 12 months; here, IPV was evaluated as an exposure. The multivariate model was adjusted for sociodemographic factors associated with both IPV and the HIV care outcomes at 12 months, in both the univariate model as well as in the literature.

All analyses were performed using Stata version 17.0 [30].

## Results

**Fig. 1:**
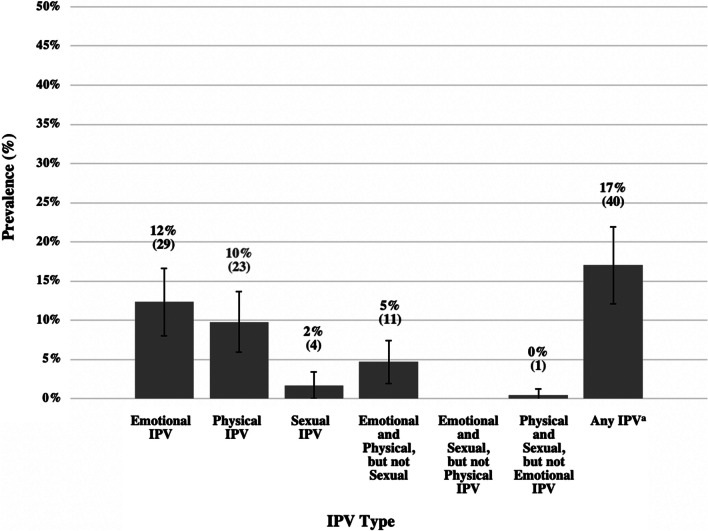
Past-year Intimate Partner Violence (IPV) Prevalence by Type, among Female Participants of the Simplifying HIV TREAtmeant and Monitoring (STREAM) Study, at baseline (N = 235), with 95% Confidence Intervals. *Created in Excel 16.69.1*. Any IPV includes any combination of emotional, physical, and/or sexual IPV

There were 235 women living with HIV (WLHIV) enrolled in the study. At baseline, the median age of the entire cohort was 30 years (Interquartile range 26–37); almost 99% identified as Black women. Table 1 presents demographic information on those who reported past-year IPV and those who did not. Of the 235 WLHIV, 19% (44) were 18–24 years old. Almost all (95%) had achieved a secondary or tertiary level of education, 83% (194) had at least one child, and 57% (133) were employed. Forty (17%) women reported experiencing any physical, sexual and/or emotional intimate partner violence (IPV) in the past year. When disaggregated by IPV type, n = 29 (12%), n = 23 (10%), and n = 4 (2%) women reported a history of past-year emotional, physical, or sexual violence, respectively (Fig. 1).

**Table 1:** Descriptive Statistics of Baseline Characteristics of Female Participants of the Simplifying HIV TREAtmeant and Monitoring (STREAM) Study, Categorized by Past-Year Intimate Partner Violence (IPV) Status (N = 235)

Variables	IPV (N = 40) n (%)	No IPV (N = 195) n (%)	p-value
*Demographics at baseline*			
Age			
> 25 years	27 (68)	164 (84)	0.014**
18–24 years	13 (33)	31 (16)	
Educational attainment			
Secondary, tertiary school	34 (85)	189 (97)	0.002***
None, primary school only	6 (15)	6 (3)	
Number of children			
> 1 child	29 (73)	165 (85)	0.066*
None	11 (28)	30 (15)	
Primary income source			
Any employment (part-time, full-time, self-employed)	24 (60)	109 (56)	0.633
No income or other support (social grants, family support, student support)	16 (40)	86 (44)	
Monthly income level among participants reported with any type of income (N = 233)			
< R1000	21 (53)	85/193 (44)	0.467
R1000-R4000	16 (40)	86/193 (45)	
R4001-R8000	1 (3)	16/193 (8)	
> R8001	2 (5)	6/193 (3)	
Harmful drinking^a^			
No	29 (73)	167 (86)	0.042**
Yes	11 (28)	28 (14)	
Depression^b^			
No	38 (95)	191 (98)	0.281
Yes	2 (5)	4 (2)	
Reported stable partner			
No	7 (17)	46 (24)	0.401
Yes	33 (83)	149 (76)	
Disclosed HIV diagnosis to partner/spouse, if reported stable partner (N = 182)			
No	3/33 (9)	45/149 (30)	0.013**
Yes	30/33 (91)	104/149 (70)	
Disclosed HIV diagnosis to partner/spouse, if reported no stable partner (N = 53)			
No	7/7 (100)	43/46 (93)	0.487
Yes	0	3/46 (7)	
Partner HIV Status, if reported stable partner (N = 142)			
HIV-negative	5/27 (19)	32/115 (28)	0.321
HIV-positive	22/27 (81)	83/115 (72)	
Contraceptive use			
No	37 (93)	186 (95)	0.450
Yes	3 (7)	9 (5)	

^a^Harmful drinking is determined by AUDIT-C score ≥ 3

^b^Depression is determined by PHQ-2 score ≥ 3

*p < 0.1

**p < 0.05

***p < 0.01

Among the women who reported any past-year IPV, 13 of 40 (33%) women were 18–24 years old, 6 (15%) had low educational attainment, 29 (73%) had at least one child, and 24 (60%) were employed. In the group that reported no past-year IPV, 31 of 195 (16%) women were 18–24 years old, 6 (3%) had low educational attainment, and 109 (56%) were employed.

Among women who reported past-year IPV, 28% were identified with concerns for harmful drinking. 93% did not use contraceptives at baseline, and 5% had potentially clinically relevant symptoms of depression. In the group of 195 women that reported no past-year IPV, 14% screened positive for harmful alcohol use, 95% did not use contraception at baseline and 2% screened as having potentially relevant symptoms of depression.

Among all WLHIV, 182 reported having a stable partner at baseline. Of those with a stable partner who also reported past-year IPV, 30 (91%) had disclosed their HIV status, compared to 104 (70%) of those who did not report past-year IPV. A high reported prevalence of women were in seroconcordant relationships; 81% (22) of women with past-year IPV who also reported having a stable partner, had partners that were also living with HIV, compared to 72% (83) of women without past-year IPV who also reported having a stable partner.

Table 2 presents the prevalence of HIV health outcomes at baseline and after 12 months of study follow-up. At baseline, a greater proportion of the cohort were not virally suppressed. However, at 12 months of study follow-up, a greater proportion had achieved viral load suppression. The primary study outcome was a composite outcome of viral load suppression and retention in care at 12 months. Of those who reported past-year IPV, 11 of 38 (29%) did not achieve the primary composite outcome. Within the group that did not report past-year IPV, 29 of 194 (15%) did not achieve the composite outcome, a 14-percentage point difference. At 12 months, 12% (4 of 34) of women who reported past-year IPV did not achieve viral suppression compared to 6% (11 of 187) among those who did not report past-year IPV.

**Table 2:** Descriptive Statistics of HIV Health Outcomes at Baseline and Study Exit (12 months) of Female Participants of the Simplifying HIV TREAtmeant and Monitoring (STREAM) Study, Categorized by Past-Year Intimate Partner Violence (IPV) Status (N = 235)

Variables	IPV (N = 40) n (%)	No IPV (N = 195) n (%)	p-value
*HIV health outcomes at baseline*			
Viral load			
Suppressed (≤ 200 copies per mL)	12 (30)	61 (31)	0.873
Not suppressed (> 200 copies per mL)	28 (70)	134 (69)	
CD4 count			
< 500	20 (50)	91 (47)	0.700
> 500	20 (50)	104 (53)	
Number of ART doses missed in last 4 days			
None	32 (80)	165 (85)	0.470
> 1 dose	8 (20)	30 (15)	
Time from testing HIV + to ART initiation (N = 231)			
< 6 months	30 (75)	122/191(64)	0.177
> 6 months	10 (25)	69/191 (36)	
*HIV health outcomes at study exit (12 months)*			
Primary outcome (VL suppression and retention in care^a^)^b^ (N = 232)			
VL suppressed (< 200 copies per mL) and retained in care	27/38 (71)	165/194 (85)	0.037**
Neither virally suppressed (> 200 copies per mL), nor retained in care	11/38 (29)	29/194 (15)	
Viral load (N = 221)			
Suppressed (≤ 200 copies per mL)	30/34 (88)	176/187 (94)	0.210
Not suppressed (> 200 copies per mL)	4/34 (12)	11/187 (6)	
Retained in care^a^ (N = 234)			
Yes	32 (80)	173/194 (89)	0.109
No	8 (20)	21/194 (11)	

^a^Retention in care is defined as documented pick-up of antiretroviral therapy (ART) within the 12-month visit window

^b^Primary study outcome

^*^p < 0.1

^**^p < 0.05

^***^p < 0.01

Table 3 presents univariate and multivariate logistic regression analyses of factors associated with IPV at baseline. The independent variables for this baseline cross-sectional logistic regression model included both sociodemographic and health-related factors; the dependent variable was a history of past-year IPV reported at baseline. The multivariate model was adjusted for age, educational attainment, number of children, harmful drinking, and disclosure of HIV status to partner. In the multivariate model, the following characteristics were associated with higher odds of past-year IPV: having only a primary school education or less (adjusted prevalence odds ratio (aPOR) 10.26, 95% Confidence Interval (CI) 2.48–42.39), having no children (aPOR 3.12, 95% CI 1.04–9.34), and harmful drinking (aPOR 3.12, 95% CI 1.04–8.42). Among 182 women with a stable partner, the odds of IPV was increased among women who had disclosed their HIV status (aPOR 4.35, 95% CI 1.17–16.10), at baseline. Being younger (18–24 years) was associated with higher odds of past-year IPV in univariate analysis (POR 2.55, 95% CI 1.19–5.47), but not in the multivariate model. There was no association between income, contraceptive use, partner’s HIV status, or a positive depression screen at baseline and past-year IPV.

**Table 3:** Factors Associated with Past-Year IPV at Baseline of Female Participants of the Simplifying HIV TREAtmeant and Monitoring (STREAM) Study (N = 235)

Variables	Unadjusted prevalence odds ratio (95% CI)	p-value	Adjusted prevalence odds ratio^a^ (95% CI)	p-value
*Demographics at baseline*				
Age				
> 25 years	REF		REF	
18–24 years	2.55 (1.19, 5.47)	0.017**	1.59 (0.55, 4.65)	0.394
Educational attainment				
Secondary, tertiary school	REF		REF	
None, primary school only	5.56 (1.69, 18.25)	0.005***	10.26 (2.48, 42.39)	0.001***
Number of children				
> 1 child	REF		REF	
None	2.09 (0.94, 4.62)	0.070*	3.12 (1.04, 9.34)	0.042**
Primary income source				
Any employment (part-time, full-time, self-employed)	REF		–	
No income or other support (social grants, family support, student support)	0.84 (0.42, 1.69)	0.634	–	–
Monthly income level among participants reported with any type of income (N = 233)				
< R1000	REF		–	
R1000–R4000	0.75 (0.37, 1.54)	0.438	–	–
R4001–R8000	0.25 (0.03, 2.02)	0.194	–	–
> R8001	1.35 (0.25, 7.17)	0.725	–	–
Harmful drinking^b^				
No	REF		REF	
Yes	2.26 (1.02, 5.04)	0.046**	3.12 (1.04, 8.42)	0.019**
Depression^c^				
No	REF		–	
Yes	2.51 (0.44, 14.2)	0.297	–	–
Disclosed to partner, if reported stable partner (N = 182)				
No	REF		REF	
Yes	4.33 (1.26, 14.91)	0.020**	4.35 (1.17, 16.10)	0.028**
Partner HIV Status, if reported stable partner (N = 142)				
HIV-negative	REF		–	
HIV-positive	1.70 (0.59, 4.86)	0.325	–	–
Contraceptive use (at or prior to baseline)				
No	REF		–	
Yes	1.68 (0.43, 6.49)	0.75	–	–

^a^Adjusted for age, educational attainment, children, harmful drinking, and disclosure of HIV status to partner

^b^Harmful drinking is determined by AUDIT-C score ≥ 3

^c^Depression is determined by PHQ-2 score ≥ 3

*p < 0.1

**p < 0.05

***p < 0.01

Table 4 presents univariate and multivariate logistic regression analyses of associations between past-year IPV and HIV care outcomes at 12 months. The independent variable for this longitudinal cohort logistic regression model was past-year IPV; the dependent variable for the primary analysis was a composite of VL suppression and retention in care at 12 months. We also present analyses of the secondary outcomes of viral suppression and retention in care.

**Table 4:** Associations between Past-Year IPV Status at Baseline and HIV Care Outcomes of Female Participants of the Simplifying HIV TREAtmeant and Monitoring (STREAM) Study, at 12 months (N = 235)

HIV health outcomes at study exit (12 months)	Unadjusted prevalence odds ratio (95% CI)	p-value	Adjusted prevalence odds ratio^a^ (95% CI)	p-value
Composite outcome (VL suppression and retention in care)^b^				
VL suppressed (≤ 200 copies per mL) and retained in care	REF		REF	
Neither virally suppressed (> 200 copies per mL), nor retained in care	2.32 (1.04, 5.18)	0.041**	1.46 (0.51, 4.15)	0.482
Viral load at exit				
Suppressed (≤ 200 copies per mL)	REF		–	–
Not suppressed (> 200 copies per mL)	2.13 (0.64, 7.14)	0.219	–	–
Retained in care				
Yes	REF			
No	2.06 (0.84, 2.05)	0.115	1.28 (0.39, 4.19)	0.679

^a^Adjusted for age, educational attainment, harmful drinking, and disclosure of HIV status to partner

^b^Primary study outcome

*p < 0.1

**p < 0.05

***p < 0.01

Experiencing past-year IPV was associated with higher odds of not achieving the primary composite HIV outcome at 12 months (aPOR 2.32, 95% CI 1.04–5.18, p = 0.041) in the univariate model, but not in the multivariate model, which was adjusted for age, educational attainment, harmful drinking, and disclosure of HIV status to partner. Past-year IPV was not associated with viral load suppression at 12 months in univariate.

## Discussion

Our findings suggest that past-year IPV was associated with an increased odds of not having achieved the primary outcome in univariate analysis; however, this association was not significant in the adjusted model, despite a 14-percentage point difference in descriptive analysis.

Women who reported past-year IPV at baseline were less likely to be retained in the study, with a nine-percentage point difference, compared to those who did not report past-year IPV. Past-year IPV was also not associated with other HIV-specific health outcomes in our cohort, like baseline VL, CD4 count, or self-described adherence to ART.

The literature is less clear on the predictive value of IPV history (recent and distant) on primary HIV-health outcomes. A meta-analysis of thirteen cross-sectional studies by Hatcher and colleagues presented some evidence to support associations between IPV and important HIV care continuum outcomes, such as lower ART use (OR 0.79, 95% CI 0.64–0.97) in five studies and lower odds of VL suppression (OR 0.64, 95% CI 0.46–0.90) in six studies [13]. A key limitation of the studies included in this meta-analysis was a lack of longitudinal results, which prevents conclusions regarding the causal relationship between IPV and engagement in care outcomes [13]. Despite these limitations, qualitative research has also supported associations between adherence and IPV [31].

Irrespective of the potential direct impact of IPV on HIV outcomes for WLHIV and engaged in care, other longitudinal studies highlight IPV as an independent cause of non-HIV related morbidity and mortality, including among WLHIV [1, 32, 33]. Our study’s findings may be limited by a small sample size, and a cohort that is well-educated, older, and participating in a clinical study, factors which may have supported adherence in this group. However, these limitations do not minimize the importance of addressing IPV experienced by WLHIV, or the urgency of integrating IPV-care into the HIV care continuum to address the needs of WLHIV.

Our study also suggests an association between disclosure of HIV status to a regular partner and past-year IPV experience. In general, research has suggested that women who disclose their HIV-positive status to male partners are more likely to experience IPV [34–37], though other studies have demonstrated increased risk of violence in the setting of *non-disclosure* [8, 38, 39]. Our study findings around disclosure also support the need for the development of more robust interventions to support WLHIV in disclosure of their status, with an emphasis on harm reduction and violence prevention. A recent systematic review found little in the way of evidence regarding interventions that could support safer disclosure [40].

We expected to observe an association between depression and IPV, as has been described in the literature, although the evidence is mixed in these models [41]. However, this finding was not observed in our cohort. Very few participants screened positively for depression; this is possibly due to stigma around disclosure of mental health issues. In terms of harmful drinking, in this study, we did observe an association between past-year IPV and harmful drinking. This has been shown in other studies to increase a woman’s susceptibility to both violence and HIV acquisition [42, 43], as well as poor outcomes for WLHIV [36]. Harmful drinking is also a modifiable risk factor; focusing on substance use among WLHIV and engaged in care is an important component of optimizing health outcomes.

Lastly, the burden of IPV in our cohort is high, with 17% reporting this in the past year. IPV was also associated with a variety of demographic factors that are also supported by the literature. Poorer women with lower educational attainment have an increased risk of both prior and future IPV [44]; studies have demonstrated a *u-shaped* relationship between educational attainment and IPV risk, with higher risk at the extremes of educational attainment [45], though with protective effects as education increases, generally [46]. We also found an association between having no children and increased risk of past-year IPV, which remained associated in multivariate analysis. WLHIV with children, especially during pregnancy and the immediate postpartum period have been shown to have a *higher* risk of experiencing violence [47–49]. Younger women are also generally at greater risk of both HIV and IPV [50], and though younger age was initially associated with IPV, this association was not sustained in the multivariate model. Screening for IPV is common in clinical settings; we recommend using the World Health Organization’s Violence Against Women questionnaire in settings where WLHIV receive care. Our study has identified WLHIV who may benefit most from targeted psychosocial support to navigate the emotional and psychological impact of IPV and to prevent future abuse while living with HIV.

Integration of IPV services into the HIV/AIDS continuum of care is rare both regionally and globally. Integrating IPV screening and management into HIV services therefore presents an opportunity to mitigate the harmful effects of IPV among WLHIV. Recognizing and addressing the unique influence of IPV-specific trauma on women living with HIV is a significant human rights and public health issue.

### Strengths and Limitations

Altogether, our study focuses on individuals already engaged in HIV care, and does not include women who may have been prevented from engaging in HIV care due to IPV. However, studies such as ours, which address risk factors for IPV among WLHIV within the HIV-care continuum are also limited.

Our study is limited by a small sample size which meant we had reduced power to detect associations between IPV and HIV related health outcomes. Participants in our study are unlikely to be truly representative of the population of WLHIV, as our population was largely urban and enrolled in a randomized control trial that enabled consistent follow-up and engagement in care, with measures to prevent attrition. Though analysis of baseline data was cross-sectional, preventing claims of causality, we used follow-up data to assess HIV outcomes, which is a concomitant strength of this analysis. Additionally, the sequence of certain events in these women’s lives is unknown, such as whether disclosure of HIV status to partner occurred prior to an experience of IPV, or vice a versa. It is possible that a population of women with a more chronic experience of IPV denotes a greater magnitude of exposure, or that a population with increased severity of IPV, with greater prevalence of sexual IPV, for example, may experience worse HIV-health outcomes. Lastly, it is important to reiterate the high prevalence of IPV in our cohort; we believe that the true value is likely higher than reported. Much has been presented elsewhere about the challenges of studying sensitive topics [51]; self-reporting often results in an underestimate of the true scope of the issue.

## Conclusion

In our setting, WLHIV reported a high burden of recent IPV, which was greater among young women without children, those with low educational attainment, those with harmful alcohol use, and those who had disclosed their HIV status to their partner. Prospective studies are needed to clarify the relationship between past-year IPV and longitudinal HIV outcomes. Altogether, identifying and addressing IPV among all WLHIV is crucial and may aid in achieving the 95–95–95 HIV diagnosis and treatment targets.

## Data Availability

Participant data for the STREAM study will be shared and de-identified (text, tables, figures, and appendices) as requested. Data will be available after the proposed use has been approved by an independent review committee.

## Code Availability

Associated code for any analyses will be shared, as requested.
